# Small RNAs OmrA and OmrB promote class III flagellar gene expression by inhibiting the synthesis of anti-Sigma factor FlgM

**DOI:** 10.1080/15476286.2020.1733801

**Published:** 2020-03-05

**Authors:** Cédric Romilly, Mirthe Hoekzema, Erik Holmqvist, E. Gerhart H. Wagner

**Affiliations:** Department of Cell and Molecular Biology, Biomedical Center, Uppsala University, Uppsala, Sweden

**Keywords:** Small RNAs, motility, flagella, FlgM, post-transcriptional control, translational regulation

## Abstract

Bacteria can move by a variety of mechanisms, the best understood being flagella-mediated motility. Flagellar genes are organized in a three-tiered cascade allowing for temporally regulated expression that involves both transcriptional and post-transcriptional control. The class I operon encodes the master regulator FlhDC that drives class II gene transcription. Class II genes include *fliA* and *flgM*, which encode the Sigma factor σ^28^, required for class III transcription, and the anti-Sigma factor FlgM, which inhibits σ^28^ activity, respectively. The *flhDC* mRNA is regulated by several small regulatory RNAs (sRNAs). Two of these, the sequence-related OmrA and OmrB RNAs, inhibit FlhD synthesis. Here, we report on a second layer of sRNA-mediated control downstream of FhlDC in the flagella pathway. By mutational analysis, we confirm that a predicted interaction between the conserved 5ʹ seed sequences of OmrA/B and the early coding sequence in *flgM* mRNA reduces FlgM expression. Regulation is dependent on the global RNA-binding protein Hfq. *In vitro* experiments support a canonical mechanism: binding of OmrA/B prevents ribosome loading and decreases FlgM protein synthesis. Simultaneous inhibition of *both* FlhD and FlgM synthesis by OmrA/B complicated an assessment of how regulation of FlgM *alone* impacts class III gene transcription. Using a combinatorial mutation strategy, we were able to uncouple these two targets and demonstrate that OmrA/B-dependent inhibition of FlgM synthesis liberates σ^28^ to ultimately promote higher expression of the class III flagellin gene *fliC.*

## Introduction

**Figure 1.**
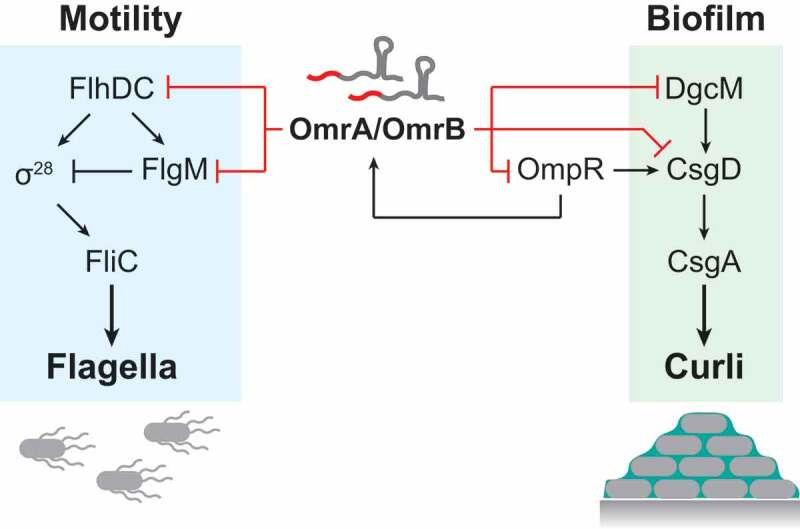
Effects of OmrA and OmrB in the regulatory networks for motile and sessile lifestyles in *E. coli.*

Bacteria are masters of rapid adaptation to changing environmental conditions. For instance, enterobacteria such as *Escherichia coli* and *Salmonella enterica* change their lifestyle in response to, e.g. nutritional status to enter a sessile (biofilm) or motile (flagellated) state. These states are generally considered mutually exclusive, which is reflected by complex layers of transcriptional control that promote the establishment of one state while simultaneously prohibiting the other [1–3]. In these decisions, the transcriptional activators FlhDC and CsgD are the master regulators of flagellar and biofilm (curli/cellulose) genes, respectively (Figure 1). Transcriptional control is additionally modulated by the levels of the second messenger cyclic-di-GMP, determined by the opposing activities of diguanylate cyclases (DGCs) and phosphodiesterases (PDEs), and by upstream acting transcription factors (TFs) such as MlrA, OmpR, and the stress/stationary Sigma factor σ^S^ (for reviews on these topics, see: [2,4-7]). On top of transcriptional control, a second, post-transcriptional level involves a large suite of small RNAs/sRNAs (OmrA, OmrB, McaS, RprA, GcvB, RydC, ArcZ, and OxyS) that, via direct regulation of FlhDC and CsgD, affect biofilm and/or flagellar gene regulation [8–14].

In the biofilm pathway, OmrA/B target three genes, *csgD, ompR*, and *dgcM* (Figure 1) [10,15–17]. CsgD is the TF responsible for activating *csgA* and *csgB*, encoding the structural proteins of curli fibers. OmpR is the response regulator of the two-component system EnvZ/OmpR, induced under osmotic stress, and a direct activator of *csgD* expression [18,19]. OmpR also activates transcription of *omrA/B*, thereby forming a negative feedback loop [16]. DgcM is a DGC that activates the TF MrlA [20], which in turn activates transcription of *csgD*. Though the same sequence motif in the 5ʹ tails of the sRNAs is used for base-pairing to all mRNA targets, the molecular mechanisms of control differ. Canonical translational inhibition works on *ompR* mRNA, where sRNAs compete against initiating ribosomes [16]. On *csgD* mRNA, both sRNAs bind far upstream of the ribosome binding site (RBS) to prevent translation initiation by an as yet unknown mechanism [10,21]. Regulation of *dgcM* mRNA involves Hfq-dependent structure remodelling to facilitate OmrA/B binding, rather than the more common matchmaking activity of this protein [17]. Ultimately, downregulation of these three genes entails inhibition of both curli formation and synthesis of cellulose. Thus, OmrA and OmrB are negative factors for a sessile lifestyle. However, both sRNAs can also inhibit motility by repressing translation of FlhD, the activator of the flagellar gene cascade [9]. This suggests a complex post-transcriptional pattern of regulation potentially affecting *both* a sessile and a motile lifestyle. In addition, these sRNAs inhibit expression of outer membrane proteins [15,16,22,23]. Thus, OmrA/B-dependent targeting of multiple mRNAs connects various regulatory networks to coordinate bacterial physiology and behaviour.

The bacterial flagellum is a complex organelle that requires coordinated temporal expression of more than 60 genes in multiple operons [24–26]. These genes are organized in a hierarchical cascade (class I–III), with several checkpoints to ensure that flagellar components are expressed and assembled sequentially [24,27,28]. The *flhDC* operon is at the top of this cascade and encodes the hetero-multimeric activator FlhD_4_C_2_ that drives σ^70^-dependent transcription of class II genes [29]. Expression of *flhDC* is tightly controlled at the transcriptional level by more than 10 TFs, and post-transcriptionally by several sRNAs and RNA-binding proteins [2,9,14,26]. Class II gene expression entails assembly of the flagellar motor, often referred to as the hook basal body (HBB). Class III genes are activated by the class II-encoded flagellar Sigma factor, σ^28^ (encoded by *fliA*), which initially is neutralized by the anti-Sigma factor FlgM [30] (Figure 1). Upon HBB completion, which results in FlgM secretion [31,32], the liberated σ^28^ activates transcription of the class III genes, including *fliC* that forms the flagellar filament [24]. σ^28^ activity is also linked to FlgM-mediated proteolysis control; FlgM binding not only inhibits σ^28^ activity but also protects it from degradation by the Lon protease [33]. Degradation of free σ^28^, once the HBB is complete and FlgM secreted, ensures that class III gene expression is restricted in time to prevent the aberrant formation of flagellar filaments [24].

Despite the extensive study of bacterial motility, the flagellar pathway still holds surprises. By now, post-transcriptional control mediated by sRNAs is known to complement transcriptional regulation of both flagellar and biofilm genes. In the present study, we identified the anti-Sigma factor-encoding *flgM* mRNA as yet another sRNA target (Figure 1). By translational inhibition of *flgM* mRNA, OmrA and OmrB help to promote class III flagellar gene expression.

## Results

### OmrA and OmrB directly inhibit FlgM synthesis by an Hfq-dependent antisense mechanism

**Figure 2.**
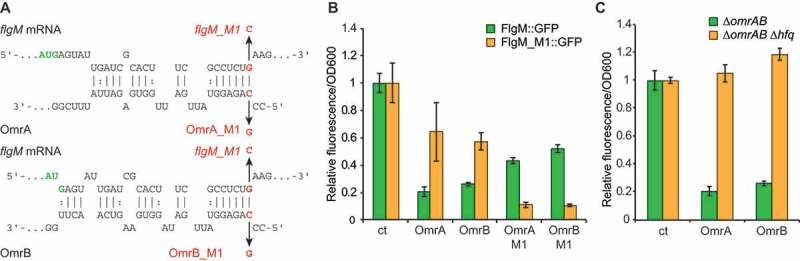
OmrA and OmrB cause direct translational inhibition of *flgM* mRNA *in vivo.*

Since sRNAs often target several genes in the same pathway [34,35], we hypothesized that OmrA/B may, in addition to *flhDC*, target other motility-associated mRNAs. Indeed, the IntaRNA algorithm [36,37] predicted base–pair interactions between OmrA/B and the *flgM* mRNA; the 5ʹ region of OmrA is complementary to the sequence encompassing codons four to nine of the *flgM* open reading frame (ORF), with codons one to nine matching OmrB (Figure 2(a)).

To assess whether OmrA/B affect *flgM* expression *in vivo*, we measured fluorescence of cells harbouring a plasmid encoding a *flgM::gfp* translational fusion in the presence or absence of either of the sRNAs; expression is driven by P_LTet0-1_ [38] followed by nucleotides +1 to 105 of *flgM* (relative to transcription start site) joined in frame with *gfp* after the first 23 codons of FlgM. Congruent with the predicted antisense interaction, overexpression of OmrA or OmrB resulted in five-fold reduced fluorescence, indicating strong repression of FlgM synthesis (Figure 2(b)). Introduction of a single point mutation (M1; Figure 2(a)) in the predicted interaction site in OmrA, OmrB, or *flgM* mRNA, reduced OmrA/OmrB-mediated repression (Figure 2(b)). Compensatory mutations designed to re-establish OmrA/B base-pairing with the *flgM* mRNA indeed restored regulation (Figure 2). The position of the OmrA/B target site in the *flgM* mRNA is further supported by *in vitro* footprinting experiments (Fig. S1). Taken together, these results strongly indicate that OmrA and OmrB inhibit FlgM synthesis by binding to the early coding region of the *flgM* mRNA.

The RNA chaperone Hfq is required for the regulatory activity of most *trans*-encoded sRNAs in enterobacteria [35,39]. Hfq stabilizes sRNAs (e.g. OmrA and OmrB levels are strongly reduced in absence of Hfq [10]), but also acts as a matchmaker platform to promote sRNA–mRNA interaction [35,39–42]. To investigate whether regulation of *flgM* by OmrA and OmrB requires Hfq, a ∆*hfq* strain was transformed with the *flgM::gfp* translational fusion and OmrA/B-overexpressing vectors. As expected, the *hfq* deletion totally abolished the sRNA-mediated repression of *flgM::gfp* (Figure 2(c)), confirming that Hfq is required for regulation.

### OmrA and OmrB inhibit FlgM translation by competing for ribosome binding

**Figure 3.**
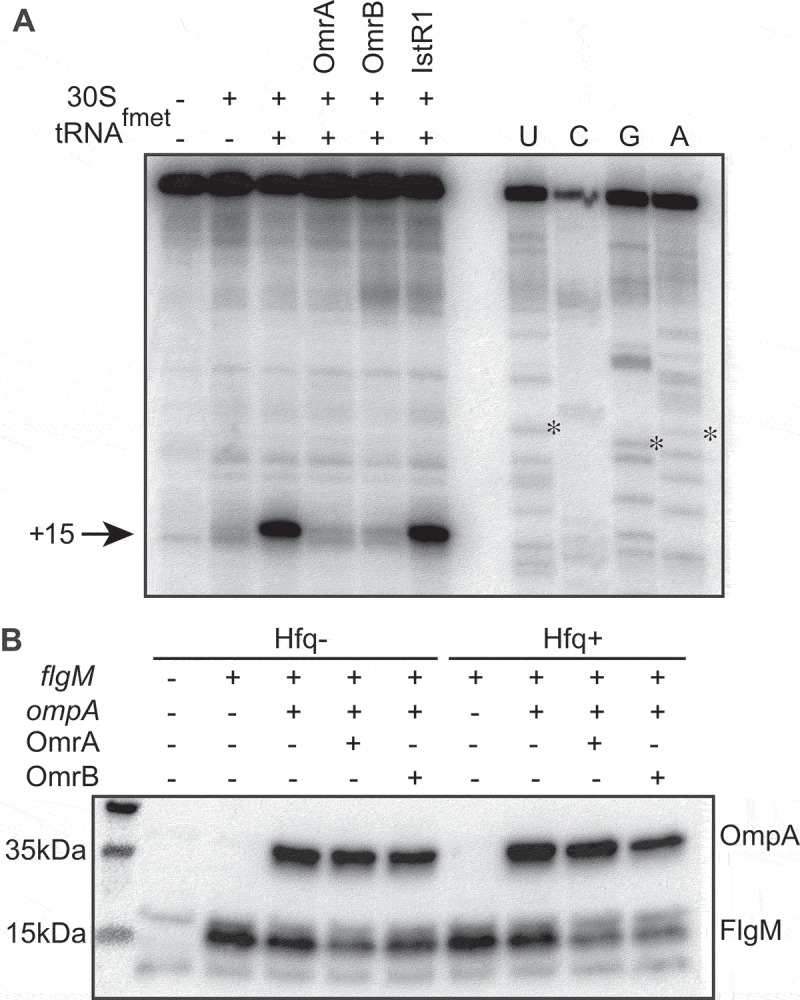
Binding of OmrA and OmrB inhibit translation of *flgM* mRNA.

The binding of OmrA/OmrB close to the AUG start codon on *flgM* mRNA suggested that repression of FlgM synthesis involves inhibition of translation initiation [43]. We performed a toeprint analysis to evaluate whether formation of the 30S initiation complex (30S-IC) on *flgM* mRNA was affected by the sRNAs. As expected, the presence of both 30S and tRNA^fmet^ resulted in a reverse transcription stop 15 nucleotides downstream of the *flgM* start codon, indicative of stable 30S-IC formation [44,45] (Figure 3(a)). Addition of either OmrA or OmrB completely abolished the toeprint signal. This strong repression is specific; addition of the unrelated sRNA IstR1 [46,47] did not affect 30S-IC formation.

OmrA/OmrB-dependent inhibition of *flgM* translation was further validated using an *in vitro* translation assay. Translation rates of *flgM-3xflag* and *ompA-3xflag* mRNAs (the latter was an internal, non-target control) were monitored by Western blot using anti-FLAG antibodies. Uponaddition of OmrA or OmrB, the accumulation of the FlgM-3xFLAG protein decreased slightly, with OmrA being more effective than OmrB, as also seen on other targets [10] (Figure 3(b)). However, in the presence of Hfq, both sRNAs strongly repressed FlgM-3xFLAG synthesis.

Altogether, the *in vitro* data corroborate the *in vivo* results on *flgM* regulation by OmrA and OmrB, suggesting direct translational inhibition via sRNA base-pairing. Moreover, the stronger inhibition observed in the presence of Hfq suggests that, in the context of active translation, Hfq facilitates sRNA–mRNA interaction.

### Mutations in OmrA abolish regulation of FlhD while supporting maintained FlgM control

**Figure 4.**
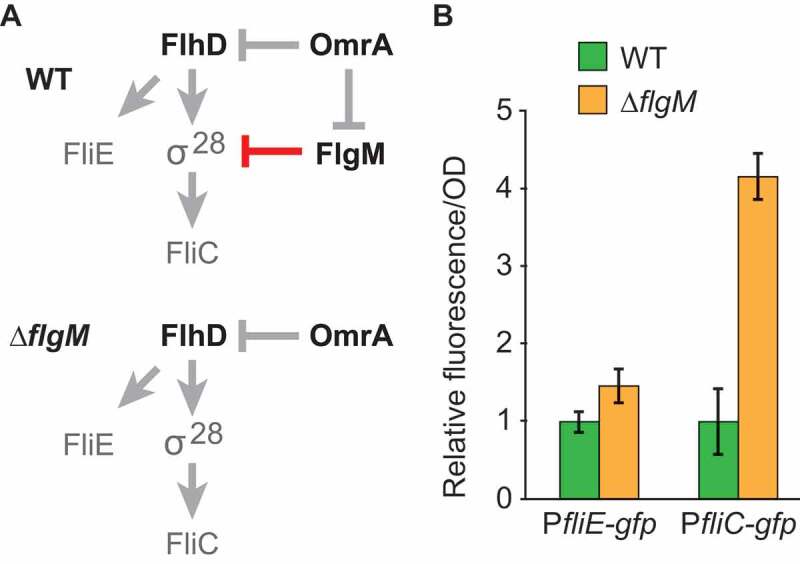
In-frame deletion of *flgM* affects class III (*fliC*) but not class II (*fliE*) flagellar gene expression.

OmrA/B inhibits synthesis of both FlhD [9] and FlgM (Figures 1–3). With respect to flagellar class II and class III genes, these regulations should have distinct effects (see Figure 4(a)). Inhibition of FlhD should reduce expression of class II genes and, indirectly via σ^28^, of class III genes. In contrast, inhibition of FlgM should promote class III transcription by increasing the cellular concentration of active σ^28^, but have little or no effect on class II genes [32,33]. To test this, we first investigated the effect of FlgM on class II and class III genes. The motile *E. coli* strain BW25113 and an isogenic strain carrying a *flgM* in-frame deletion were transformed with plasmids carrying transcriptional fusions between the promoters of *fliE* (class II) or *fliC* (class III) and *gfp*. As expected, the *fliC* promoter was strongly activated in the ∆*flgM* strain compared to wild type, whereas the absence of FlgM had no significant effect on *fliE* promoter activity (Figure 4(b), Fig. S2). These results are in line with previous reports showing that FlgM-mediated control of σ^28^ specifically impacts class III genes [25,32].

**Figure 5.**
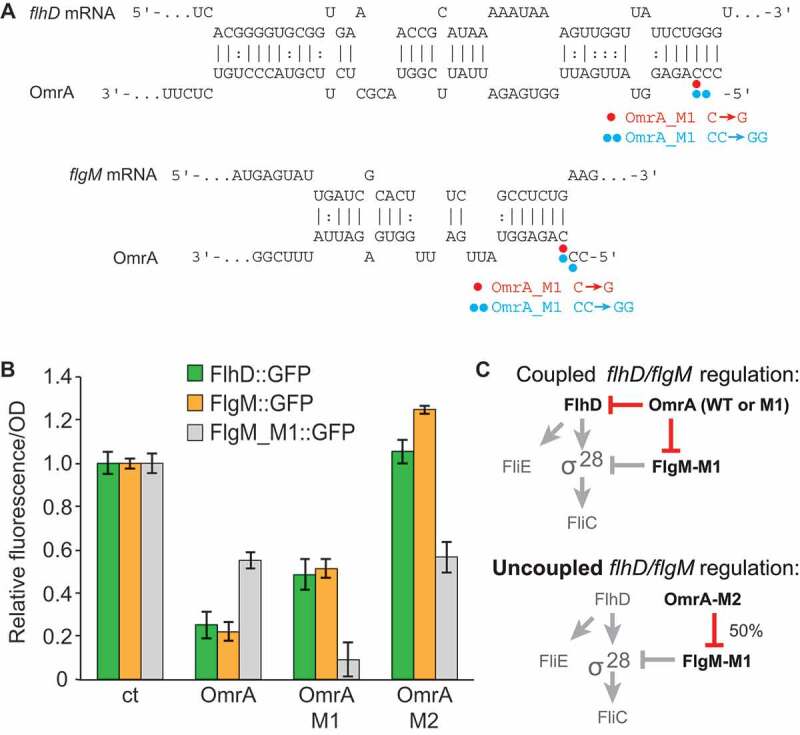
Uncoupled regulation of *flhD* and *flgM*_*M1* by mutant versions of OmrA.

Next, we sought to construct a regulatory circuit in which we could analyse the effects of OmrA/B-dependent regulation of FlgM, and its consequences for class III transcription, in the absence of OmrA/B’s effect on FlhD. We first tested how OmrA/B and mutants thereof affected the expression from a translational *flhD::gfp* fusion. While wild-type sRNAs and M1 mutant RNAs (see Figure 2(b)) repressed *flhD::gfp* expression, a second mutant variant, M2 (CC to GG mismatch at positions 2 and 3 of OmrA/B; Figure 5(a)), failed to do so (Figure 5(b), Fig. S3). Moreover, OmrA-mediated inhibition of FlhD translation was stronger than that of OmrB (Fig. S2). Since this paralleled the stronger OmrA effect on FlgM in the *in vitro* translation assay (Figure 3(b)), we focused on OmrA-mediated regulation of flagellar genes from now on. We monitored the effect of the same OmrA mutant RNAs, M1 and M2, on expression from the target site-mutated translational *flgM_M1::gfp* fusion. OmrA_M1 strongly repressed *flgM_M1::gfp* translation, while both the wild-type OmrA and the mutant OmrA_M2 maintained about two-fold repression (Figure 5(b)). Thus, a circuit in which *flgM* carries the M1 mutation, and OmrA carries the M2 mutation, allows uncoupling of the OmrA-dependent regulation of FlhD and FlgM (Figure 5(c)). This in turn enabled us to specifically monitor the consequences of OmrA-dependent regulation of FlgM alone on flagellar gene expression.

### Repression of FlgM synthesis by OmrA/B increases class III flagellar gene expression

**Figure 6.**
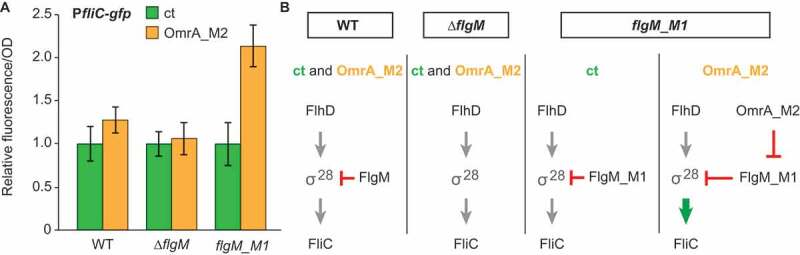
OmrA-mediated regulation of *flgM* promotes class III flagellar gene expression.

To understand how OmrA/B-mediated regulation of *flgM* impacts class III gene expression, scarless mutagenesis was used to introduce the M1 mutation into the chromosomal *flgM* locus. Wild type, *flgM_M1*, and *∆flgM* strains were transformed with the P*fliC-gfp* transcriptional fusion plasmid together with a plasmid constitutively expressing OmrA_M2, or an empty control plasmid. In strains carrying the control plasmid, absolute fluorescence levels were comparable between wild-type and *flgM*_*M1*, indicating that the silent M1 mutation neither affects FlgM expression nor activity whereas, in contrast, the *∆flgM* strain exhibited increased fluorescence (Fig. S2). This is in agreement with Figure 4. Importantly, the strain carrying the *flgM_M1* mutation and OmrA-M2 showed two-fold higher fluorescence, compared to the strain with the control plasmid. By contrast, neither the strain with wild-type *flgM*, nor the ∆*flgM* strain, exhibited OmrA_M2-dependent effects on the *fliC* reporter (Figure 6). Since *flhD* and *flgM_M1* regulation is uncoupled in this background (Figure 5), and since FlgM and FlgM_M1 proteins are similarly active, we conclude that OmrA/B-mediated regulation of FlgM translation positively contributes to flagella expression, and/or its timing, independently of *flhD* post-transcriptional regulation by the same sRNAs.

## Discussion

In this report, we show that the *E. coli* sRNAs OmrA and OmrB negatively control the expression of the anti-Sigma factor FlgM. This frees up α^28^, thereby indirectly promoting the expression of the flagellar class III genes. *In vivo*, overexpression of either sRNA decreased fluorescence from a translational *flgM::gfp* reporter, and mutational analysis, supports that regulation is Hfq-dependent, direct, and requires an anti-sense interaction between OmrA or OmrB and the *flgM* mRNA (Figures 1 and 2). Toeprint analysis and *in vitro* translation assays suggested sRNA binding-dependent canonical translational inhibition (Figure 3). As previously reported [32], deletion of *flgM* activates a *fliC* transcriptional fusion by approximately four-fold compared to that of a wild-type strain (Figure 5). To monitor the impact of OmrA on class III gene expression only via FlgM, uncoupling of its regulatory effect on *flgM* from that on *flhD* was required. The OmrA_M2 mutant RNA could no longer regulate *flhD* (Figure 5) but still repressed a *flgM_M1* mutant mRNA, albeit at half the efficiency as that of a fully matched pair of RNAs. This experimental setup ensured that the master regulator FlhD_4_C_2_ was expressed and could initiate flagella synthesis even during constitutive OmrA_M2 expression. Hence, we could specifically monitor the effect of OmrA_M2-dependent regulation of *flgM_M1* on class III genes (Figure 5). The results confirmed that OmrA_M2 control of *flgM_M1* did indeed increase *fliC* expression (by two-fold; Figure 6). This is less than the four-fold increased expression obtained by deleting *flgM* (Figure 4), which may be explained by the only 50% inhibition of the *flgM*_M1 mRNA by OmrA_M2 (Figure 5). Taken together, OmrA/B not only repress the initial step of the flagellar pathway via FlhDC, but also activate subsequent class III gene expression via FlgM.

What is the biological role of OmrA/B-dependent indirect activation of class III flagellar genes? One rationale is stabilization of states. Keeping a biofilm program OFF, and motility ON, largely depends on layers of transcriptional control, which also counter-regulate the opposite program [1–3]. The biofilm-OFF program is reinforced by many inhibitory sRNAs converging on *csgD* expression [2,35,48]. As discussed elsewhere, silent states are difficult to maintain by transcriptional repressors [34,49,50]. Conversely, post-transcriptional inhibition by sRNAs can maintain a silent state by scavenging mRNAs that have escaped transcriptional repression [35,51]. Simultaneously, reinforcing the motility ON program also involves sRNAs, such as McaS that activates FlhD translation [14] and – as we show here – OmrA/B that repress the anti-Sigma factor FlgM. Both McaS and OmrA/B are also known inhibitors of *csgD* [14].

OmrA/B, via downregulation of the anti-Sigma factor FlgM which frees up σ^28^, act within the second tier of flagellar gene regulation, and promote an overall increase in class III flagellar gene expression. Since these sRNAs also inhibit *flhD*, which should decrease both class II and III flagellar gene expression, this may seem counterintuitive. We note however that FlhD_4_C_2_ and σ^28^ control different regulons [25,52]. The class III flagella genes require σ^28^ for expression and encode proteins required for late flagella assembly (flagellar filament and motor) as well as chemotaxis. Therefore, once flagella synthesis is initiated, OmrA/B might favour the completion of the pathway rather than the abortion of an energetically costly process. σ^28^ also drives expression of *trg, tar*, and *aer* [25]. While not classified as class III flagella genes, they are sensory proteins involved in chemotaxis. Modulation of FlhD_4_C_2_ (as well as DgcM and CsgD) levels under osmotic stress conditions, when OmrA and OmrB expression is activated via the OmpR/EnvZ two component system, could be of importance when assembly of large structures such as flagella (and curli-fibres) at the cell membrane might have detrimental effects on cell survival [53]. However, the new layer provided by the regulation of *flgM* could help the bacteria to integrate multiple environmental signals, and combine McaS and OmrA/B to coordinate and promote bacterial motility in response to osmotic and low nutrient stress. This might simultaneously boost their combined negative effect on biofilm formation, tipping the balance towards motility under specific conditions [10,14,17,21].

The experiments conducted in this paper were carried out in liquid medium. Though cell cultures faithfully recapitulate the temporal patterns of hierarchical flagellar gene expression [27,54], structured enterobacterial biofilms on solid media display features that cannot be assessed in liquid. Foremost, though cells in the biofilm structure show nutrient-gradient-depending differences in activity of the key regulators (e.g. PDEs, DGCs, σ^S^, FhlDC, CsgD), cell states in middle layers indicate microheterogeneity [54,55]. It would be an interesting question to ask whether, for instance, the balance of OmrA/B-mediated effects on *flgM*, and *fhlD*, which oppositely affect flagellar gene expression, are dependent on external or internal factors in a structured biofilm. The complexity of flagellar gene regulation, with regulatory motifs that may generate enforcement of an ongoing program but also cell-to-cell variation, remains a great challenge.

## Materials and methods

### Chemicals, reagents and oligodeoxyribonucleotides

Growth media components were purchased from Oxoid. Chemicals and oligodeoxyribonucleotides (Table S1) were bought from Sigma-Aldrich, and all reagents from ThermoFisher Scientific, unless otherwise stated. Plasmid DNA and PCR products were purified with the mini prep kit (K0503) and the PCR clean up kit (K0702) from Thermo Scientific.

### Growth conditions

Cells were routinely grown aerobically at 37°C in L Broth (5 g/L yeast extract, 10 g/L NaCl, 10 g/L tryptone). When required, antibiotics were added at 100 µg/ml (ampicillin), 50 µg/ml (kanamycin), and 15 µg/ml (chloramphenicol, tetracycline).

### Bacterial strains and plasmids

Strains and plasmids are listed in Table S2 and Table S3, respectively. Plasmids pOmrA and pOmrB were previously published [10]. Plasmid pFlgM::GFP was constructed by inserting a *Nsi*I/*Nhe*I-digested PCR product (primers EHO-671/EHO-433) into *Nsi*I/*Nhe*I-digested plasmid pXG-10 [38]. Plasmids pFlgM_M1, pOmrA_M1 and pOmrB_M1 were created by site-directed mutagenesis (QuickChange II, Stratagene), using primer pairs EHO-696/EHO697, EHO-698/EHO-699 or EHO-700/EHO-701, and plasmids pFlgM::GFP, pOmrA or pOmrB as templates, respectively. Chemically competent *E. coli* Top10 cells were used for all transformations (C404003, Invitrogen). In-frame deletions of *flgM* and *flgM_M1* were obtained using the Lambda red scarless mutagenesis method as described in [17]. For *flgM-3xflag* transcription, a plasmid with a T7 promoter, the *flgM* ORF, a *3xflag* sequence, and T7 terminator was created using the pET52-b vector from Novagen as backbone. The plasmid was linearized by PCR using primers MHO-238 and MHO-239, introducing a *3xflag* sequence and *Xho*I/*Aat*II restriction sites while simultaneously deleting the His-tag and multiple cloning site. The *flgM* 5ʹ UTR and ORF up to the stop codon were PCR-amplified with primers MHO-240/MHO-241 to introduce *Xho*I/*Aat*II restriction sites. After digestion (fast-digest, Thermo Scientific), vector and *flgM* insert were ligated using ready-to-go T4 ligase (Amersham, 27-0361-01), resulting in the p*flgM-3xflag* plasmid.

### In vitro transcription and labelling of RNA

OmrA and OmrB small RNAs were generated as previously described [10]. To generate the *flgM* mRNA fragment (nucleotides 1–200), DNA templates containing a T7 promoter sequence were generated by PCR using primers EHO-714/MHO-200 with *E. coli* MC4100 *relA*+ DNA as a template. DNA templates for *ompA-3xflag and flgM-3xflag* mRNA transcription were obtained by PCR with primers MHO-207 and MHO-230 using *E. coli* MC4100 *ompA-3xflag* (E397) as a template, and with primers MHO-244/MHO-245 with p*flgM 3xflgM* as template, respectively. The resulting PCR products were used for *in vitro* transcription using the Megascript kit (Life technologies, AM1330) according to the manufacturer’s instructions. Full-length mRNAs were purified using denaturing polyacrylamide-urea gel electrophoresis. After detection by UV-shadowing, RNA was eluted by incubation of the gel slice in elution buffer (300 mM sodium acetate, 0.1% SDS, 1 mM EDTA). After phenol-chloroform extraction, RNAs were precipitated with cold ethanol, centrifuged, and finally dissolved in sterile water. RNA concentration and quality were assessed by Nanodrop and denaturing PAGE.

5ʹ end labelling was performed on RNA pre-treated with CIAP (Invitrogen™, #18,009-019) with T4 PNK (Thermo Scientific, #EK0031) and [γ-P^32^]ATP (10 mCi/ml, 3000 Ci/mmol). Labelled RNAs were gel-purified as described above.

Before use and unless specified otherwise, RNAs were denatured separately in water for 1 min at 95°C followed by 1 min incubation on ice, and renatured 5 min at 37°C in renaturation buffer (100 mM potassium acetate, 10 mM magnesium acetate, 50 mM Tris-HCl pH 7,5).

### RNA secondary structure probing

Structural probing was carried out on 5ʹ end labelled *flgM* RNA (10 nM, 50 000 cpm) with or without OmrA/B (10 µM final concentration) at 37°C, with carrier yeast tRNA. After renaturation, mRNAs and sRNAs were incubated together for 15 min. Enzymatic probing was done with 0.1 unit of RNase T1 (Invitrogen™, AM2283) for 5 min and stopped by addition of cold sodium acetate immediately followed by phenol/chloroform/isoamyl alcohol (25/24/1) extraction and RNA precipitation. RNA pellets were dried and dissolved in loading dye. Samples were resolved by 8% denaturing urea-PAGE. The gel was fixed for 5 min at r.t. (10% ethanol, 6% acetic acid), transferred to a 3 mm Whatman filter, then dried at 80°C for 1 h. Radioactive signals were detected using PhosphorImager screens and a PMI scanner™ (Biorad).

### Toeprinting assay

Toeprinting reactions and 30S ribosome preparations were carried out as in [56]. Final concentrations of reaction components were: 20 nM mRNA, 100 nM 30S, 300 nM initiator tRNA, 0.5 mM dNTPs, and 10 µM sRNAs (OmrA/B or IstR1).

### In vitro translation assay

For *in vitro* translation assays, the PURExpress® *In Vitro* Protein Synthesis Kit (New England Biolabs, E6800 S [57]) was used. Final concentrations: 50 nM *flgM-3xflag*, 0.03 nM *ompA-3xflag*, 2.5 µM OmrA or OmrB, and 18 nM Hfq (prepared as in [58]). After renaturation, mRNAs (± Hfq and/or OmrA/B), were pre-incubated in pre-mixed PURExpress solution A and B. Translation was carried out for 30 min at 37°C, followed by addition of Laemmli sample buffer (Biorad, 1610747) supplemented with 1/10th volume of 2-mercaptoethanol. Samples were kept on ice, before boiling at 95°C for 5 min. Proteins and ladder (PageRuler™ Prestained Protein Ladder, 26616, Thermo Scientific) were separated on an Any kD™ Mini-PROTEAN® TGX Stain-Free™ Gel (Biorad, 4509036). Proteins were transferred to a Trans-Blot Turbo™ Mini PVDF membrane (Biorad, 1704156) using the Trans-Blot Turbo Transfer System (Biorad, ‘Any Kd’ preset program). After o.n. blocking in Odyssey® Blocking Buffer (PBS) (LI-COR) at 4°C, translation products were detected with monoclonal ANTI-FLAG M2-Peroxidase (HRP) antibody (Sigma, A8592). After 1 h of incubation with the antibody at r.t., membranes were washed 3x with PBS-Tween 20 (0.1%) followed by two washes with PBS. Blots were developed using Amersham™ ECL™ Prime Western Blotting Detection Reagent (GE Healthcare, RPN2109) and imaged on a ChemiDoc™ MP (Biorad). Images were analysed with Image lab software (version 4.0 built 16).

### Fluorescence measurements

Bacterial cultures grown overnight from single colonies were diluted 1:100 in fresh LB medium and grown in black 96-well plates with clear flat bottom (Costar®) at 37°C. Fluorescence (GFP: excitation 480 nm, emission 520 nm) and optical density (600 nm) were measured for 23 h at 5 min intervals in a plate reader (Tecan infinite pro). Fluorescence values were divided by optical density and corrected for media background. Background-subtracted GFP/OD_600_ from each strain and time point were averaged and expressed relative to the relevant control strain.

## Supplementary Material

Supplemental Material

## References

[1] Guttenplan SB, Kearns DB. Regulation of flagellar motility during biofilm formation. FEMS Microbiol Rev. 2013;37:849–871. 23480406 10.1111/1574-6976.12018 PMC3718880

[2] Mika F, Hengge R. Small regulatory RNAs in the control of motility and biofilm formation in E. coli and Salmonella. Int J Mol Sci. 2013;14:4560–4579. 23443158 10.3390/ijms14034560 PMC3634460

[3] Pesavento C, Becker G, Sommerfeldt N, et al. Inverse regulatory coordination of motility and curli-mediated adhesion in Escherichia coli. Genes Dev. 2008;22:2434–2446. 18765794 10.1101/gad.475808 PMC2532929

[4] Gerstel U, Römling U. The csgD promoter, a control unit for biofilm formation in Salmonella typhimurium. Res Microbiol. 2003;154:849–871. 10.1016/j.resmic.2003.08.005 14643403

[5] Gerstel U, Park C, Römling U. Complex regulation of csgD promoter activity by global regulatory proteins. Mol Microbiol. 2003;49:639–654. 12864849 10.1046/j.1365-2958.2003.03594.x

[6] Hengge R. Proteolysis of σS (RpoS) and the general stress response in Escherichia coli. Res Microbiol. 2009;160:667–676. 19765651 10.1016/j.resmic.2009.08.014

[7] Povolotsky TL, Hengge R. ‘Life-style’ control networks in Escherichia coli: signaling by the second messenger c-di-GMP. J.Biotechnol. 2012;160:10–16. 22226726 10.1016/j.jbiotec.2011.12.024

[8] Bordeau V, Felden B. Curli synthesis and biofilm formation in enteric bacteria are controlled by a dynamic small RNA module made up of a pseudoknot assisted by an RNA chaperone. Nucleic Acids Res. 2014;42:4682–4696. 24489123 10.1093/nar/gku098 PMC3985669

[9] De Lay ND, Gottesman S. A complex network of small non‐coding RNAs regulate motility in Escherichia coli. Mol Microbiol. 2012;86:524–538. 22925049 10.1111/j.1365-2958.2012.08209.x PMC7458410

[10] Holmqvist E, Reimegård J, Sterk M, et al. Two antisense RNAs target the transcriptional regulator CsgD to inhibit curli synthesis. Embo J. 2010;29:1840–1850. 20407422 10.1038/emboj.2010.73 PMC2885931

[11] Jørgensen MG, Nielsen JS, Boysen A, et al. Small regulatory RNAs control the multi-cellular adhesive lifestyle of Escherichia coli. Mol Microbiol. 2012;84:36–50. 22250746 10.1111/j.1365-2958.2012.07976.x

[12] Jørgensen MG, Thomason MK, Havelund J, et al. Dual function of the McaS small RNA in controlling biofilm formation. Genes Dev. 2013;27:1132–1145. 23666921 10.1101/gad.214734.113 PMC3672647

[13] Mika F, Busse S, Possling A, et al. Targeting of csgD by the small regulatory RNA RprA links stationary phase, biofilm formation and cell envelope stress in Escherichia coli. Mol Microbiol. 2012;84:51–65. 22356413 10.1111/j.1365-2958.2012.08002.x PMC3465796

[14] Thomason MK, Fontaine F, De Lay N, et al. A small RNA that regulates motility and biofilm formation in response to changes in nutrient availability in Escherichia coli. Mol Microbiol. 2012;84:17–35. 22289118 10.1111/j.1365-2958.2012.07965.x PMC3312966

[15] Guillier M, Gottesman S. Remodelling of the Escherichia coli outer membrane by two small regulatory RNAs. Mol Microbiol. 2006;59:231–247. 16359331 10.1111/j.1365-2958.2005.04929.x

[16] Guillier M, Gottesman S. The 5′ end of two redundant sRNAs is involved in the regulation of multiple targets, including their own regulator. Nucleic Acids Res. 2008;36:6781–6794. 18953042 10.1093/nar/gkn742 PMC2588501

[17] Hoekzema M, Romilly C, Holmqvist E, et al. Hfq‐dependent mRNA unfolding promotes sRNA‐based inhibition of translation. Embo J. 2019;38:e101199. 30833291 10.15252/embj.2018101199 PMC6443205

[18] Prigent-Combaret C, Brombacher E, Vidal O, et al. Complex regulatory network controls initial adhesion and biofilm formation in Escherichia coli via regulation of thecsgD gene. J Bacteriol. 2001;183:7213–7223. 11717281 10.1128/JB.183.24.7213-7223.2001 PMC95571

[19] Vidal O, Longin R, Prigent-Combaret C, et al. Isolation of an Escherichia coli K-12 mutant strain able to form biofilms on inert surfaces: involvement of a new ompR allele that increases curli expression. J Bacteriol. 1998;180:2442–2449. 9573197 10.1128/jb.180.9.2442-2449.1998 PMC107187

[20] Lindenberg S, Klauck G, Pesavento C, et al. The EAL domain protein YciR acts as a trigger enzyme in a c-di-GMP signalling cascade in E. coli biofilm control. Embo J. 2013;32:2001–2014. 23708798 10.1038/emboj.2013.120 PMC3715855

[21] Holmqvist E, Reimegård J, Wagner EGH. Massive functional mapping of a 5′-UTR by saturation mutagenesis, phenotypic sorting and deep sequencing. Nucleic Acids Res. 2013;41:e122. 23609548 10.1093/nar/gkt267 PMC3695526

[22] Brosse A, Korobeinikova A, Gottesman S, et al. Unexpected properties of sRNA promoters allow feedback control via regulation of a two-component system. Nucleic Acids Res. 2016;44:9659–9666. 10.1093/nar/gkw642 PMC5175337 27439713

[23] Jagodnik J, Chiaruttini C, Guillier M. Stem-loop structures within mRNA coding sequences activate translation initiation and mediate control by small regulatory RNAs. Mol Cell. 2017;68:158–170.e3. 28918899 10.1016/j.molcel.2017.08.015

[24] Chevance FFV, Hughes KT. Coordinating assembly of a bacterial macromolecular machine. Nat Rev Microbiol. 2008;6:455–465. 18483484 10.1038/nrmicro1887 PMC5963726

[25] Fitzgerald DM, Bonocora RP, Wade JT. Comprehensive mapping of the Escherichia coli flagellar regulatory network. PLoS Genet. 2014;10:e1004649. 25275371 10.1371/journal.pgen.1004649 PMC4183435

[26] Yakhnin AV, Baker CS, Vakulskas CA, et al. CsrA activates *flhDC* expression by protecting *flhDC* mRNA from RNase E-mediated cleavage. Mol Microbiol. 2013;87:851–866. 23305111 10.1111/mmi.12136 PMC3567230

[27] Kalir S, McClure J, Pabbaraju K, et al. Ordering genes in a flagella pathway by analysis of expression kinetics from living bacteria. Science. 2001;292:2080–2083. 11408658 10.1126/science.1058758

[28] Kutsukake K, Ohya Y, Iino T. Transcriptional analysis of the flagellar regulon of Salmonella typhimurium. J Bacteriol. 1990;172:741–747. 2404955 10.1128/jb.172.2.741-747.1990 PMC208501

[29] Wang S, Fleming RT, Westbrook EM, et al. Structure of the Escherichia coli flhdc complex, a prokaryotic heteromeric regulator of transcription. J Mol Biol. 2006;355:798–808. 16337229 10.1016/j.jmb.2005.11.020

[30] Hughes KT, Mathee K. The anti-sigma factors. Annu Rev Microbiol. 1998;52:231–286. 9891799 10.1146/annurev.micro.52.1.231

[31] Hughes KT, Gillen KL, Semon MJ, et al. Sensing structural intermediates in bacterial flagellar assembly by export of a negative regulator. Science. 1993;262:1277–1280. 8235660 10.1126/science.8235660

[32] Karlinsey JE, Tanaka S, Bettenworth V, et al. Completion of the hook–basal body complex of the Salmonella typhimurium flagellum is coupled to FlgM secretion and fliC transcription. Mol Microbiol. 2000;37:1220–1231. 10972838 10.1046/j.1365-2958.2000.02081.x

[33] Barembruch C, Hengge R. Cellular levels and activity of the flagellar sigma factor FliA of Escherichia coli are controlled by FlgM-modulated proteolysis. Mol Microbiol. 2007;65:76–89. 17537210 10.1111/j.1365-2958.2007.05770.x

[34] Beisel CL, Storz G. Base pairing small RNAs and their roles in global regulatory networks. FEMS Microbiol Rev. 2010;34:866–882. 20662934 10.1111/j.1574-6976.2010.00241.x PMC2920360

[35] Wagner EGH, Romby P. Small RNAs in bacteria and archaea: who they are, what they do, and how they do it. Adv Genet. 2015;90:133–208. 26296935 10.1016/bs.adgen.2015.05.001

[36] Busch A, Richter AS, Backofen R. IntaRNA: efficient prediction of bacterial sRNA targets incorporating target site accessibility and seed regions. Bioinformatics. 2008;24:2849–2856. 18940824 10.1093/bioinformatics/btn544 PMC2639303

[37] Mann M, Wright PR, Backofen R. IntaRNA 2.0: enhanced and customizable prediction of RNA–RNA interactions. Nucleic Acids Res. 2017;45:W435–W439. 28472523 10.1093/nar/gkx279 PMC5570192

[38] Urban JH, Vogel J. Translational control and target recognition by Escherichia coli small RNAs in vivio. Nucleic Acids Res. 2007;35:1018. 10.1093/nar/gkl1040 PMC1807950 17264113

[39] Vogel J, Luisi BF. Hfq and its constellation of RNA. Nat Rev Microbiol. 2011;9:578–589. 21760622 10.1038/nrmicro2615 PMC4615618

[40] Santiago‐Frangos A, Woodson SA. Hfq chaperone brings speed dating to bacterial sRNA. WIREs RNA. 2018;9:e1475. 29633565 10.1002/wrna.1475 PMC6002925

[41] Schu DJ, Zhang A, Gottesman S, et al. Alternative Hfq‐sRNA interaction modes dictate alternative mRNA recognition. Embo J. 2015;34:2557–2573. 26373314 10.15252/embj.201591569 PMC4609186

[42] Zhang A, Wassarman KM, Rosenow C, et al. Global analysis of small RNA and mRNA targets of Hfq. Mol Microbiol. 2003;50:1111–1124. 14622403 10.1046/j.1365-2958.2003.03734.x

[43] Bouvier M, Sharma CM, Mika F, et al. Small RNA binding to 5′ mRNA coding region inhibits translational initiation. Mol Cell. 2008;32:827–837. 19111662 10.1016/j.molcel.2008.10.027

[44] Hartz D, McPheeters DS, Traut R, et al. Extension inhibition analysis of translation initiation complexes. Methods Enzymol. 1988;164:419–425. 2468068 10.1016/s0076-6879(88)64058-4

[45] Hüttenhofer A, Noller HF. Footprinting mRNA-ribosome complexes with chemical probes. Embo J. 1994;13:3892–3901. 8070416 10.1002/j.1460-2075.1994.tb06700.x PMC395302

[46] Darfeuille F, Unoson C, Vogel J, et al. An antisense RNA inhibits translation by competing with standby ribosomes. Mol Cell. 2007;26:381–392. 17499044 10.1016/j.molcel.2007.04.003

[47] Vogel J, Argaman L, Wagner EGH, et al. The small RNA IstR inhibits synthesis of an SOS-induced toxic peptide. Curr Biol. 2004;14:2271–2276. 15620655 10.1016/j.cub.2004.12.003

[48] Boehm A, Vogel J. The csgD mRNA as a hub for signal integration via multiple small RNAs. Mol Microbiol. 2012;84:1–5. 22414234 10.1111/j.1365-2958.2012.08033.x

[49] Levine E, Hwa T. Small RNAs establish gene expression thresholds. Curr Op Microbiol. 2008;11:574–579. 10.1016/j.mib.2008.09.016 PMC2613760 18935980

[50] Levine E, Zhang Z, Kuhlman T, et al. Quantitative characteristics of gene regulation by small RNA. PLoS Biol. 2007;5:e229. 17713988 10.1371/journal.pbio.0050229 PMC1994261

[51] Holmqvist E, Wagner EGH. Impact of bacterial sRNAs in stress responses. Biochem Soc Transact. 2017;45:1203–1212. 10.1042/BST20160363 PMC5730939 29101308

[52] Zhao K, Liu M, Burgess RR. Adaptation in bacterial flagellar and motility systems: from regulon members to ‘foraging’-like behavior in Ecoli. Nucleic Acids Res. 2007;35:4441–4452. 17576668 10.1093/nar/gkm456 PMC1935009

[53] Mika F, Hengge R. Small RNAs in the control of RpoS, CsgD, and biofilm architecture of Escherichia coli. RNA Biol. 2014;11:494–507. 25028968 10.4161/rna.28867 PMC4152358

[54] Klauck G, Serra DO, Possling A, et al. Spatial organization of different sigma factor activities and c-di-GMP signalling within the three-dimensional landscape of a bacterial biofilm. Open Biol. 2018;8:180066. 30135237 10.1098/rsob.180066 PMC6119863

[55] Serra DO, Hengge R. A c-di-GMP-based switch controls local heterogeneity of extracellular matrix synthesis which is crucial for integrity and morphogenesis of Escherichia coli macrocolony biofilms. J Mol Biol. 2019;431:4775–4793. 30954572 10.1016/j.jmb.2019.04.001

[56] Romilly C, Deindl S, Wagner EGH. The ribosomal protein S1-dependent standby site in tisB mRNA consists of a single-stranded region and a 5′ structure element. Proc Natl Acad Sci USA. 2019;116:15901–15906. 31320593 10.1073/pnas.1904309116 PMC6690012

[57] Shimizu Y, Inoue A, Tomari Y, et al. Cell-free translation reconstituted with purified components. Nat Biotechnol. 2001;19:751–755. 11479568 10.1038/90802

[58] Fender A, Elf J, Hampel K, et al. RNAs actively cycle on the Sm-like protein Hfq. Genes Dev. 2010;24:2621–2626. 21123649 10.1101/gad.591310 PMC2994036

