# Supplementary material for: Small RNAs OmrA and OmrB promote class III flagellar gene expression by inhibiting the synthesis of anti-Sigma factor FlgM
Source: RNA Biol. 2020 Mar 5;17(6):872–80. doi: 10.1080/15476286.2020.1733801 (PMC7549644; doi:10.1080/15476286.2020.1733801)
Supplement: Supplemental Material [file KRNB_A_1733801_SM6017.pdf]

Small RNAs OmrA and OmrB promote class III flagellar gene expression by inhibiting the synthesis of anti-Sigma factor FlgM

Cédric Romilly, Mirthe Hoekzema[(a)], Erik Holmqvist*, and E. Gerhart. H. Wagner*

Department of Cell and Molecular Biology, Biomedical Center, Uppsala university, Box 596, Husargatan 3, S-75124 Uppsala, Sweden

(a) Current address: Department of Molecular Microbiology, Groningen Biomolecular Sciences and Biotechnology Institute, University of Groningen, Nijenborg 7, 9747 AG Groningen, The Netherlands.

*Corresponding authors: erik.holmqvist@icm.uu.se, gerhart.wagner@icm.uu.se

Supplemental material

Figure S1. OmrA and B base-pair with the *flgM* mRNA near the ribosome binding site.

Figure S2. Absolute GFP/OD600 values of the experiments presented in the main paper.

Figure S3. Duplex prediction between the *flhD, flgM* or *flgM_M1* mRNAs with OmrA, OmrA_M1, or OmrA_M2

Table S1. Primers used in this study

Table S2. Strains used in this study

Table S3. Plasmids used in this study

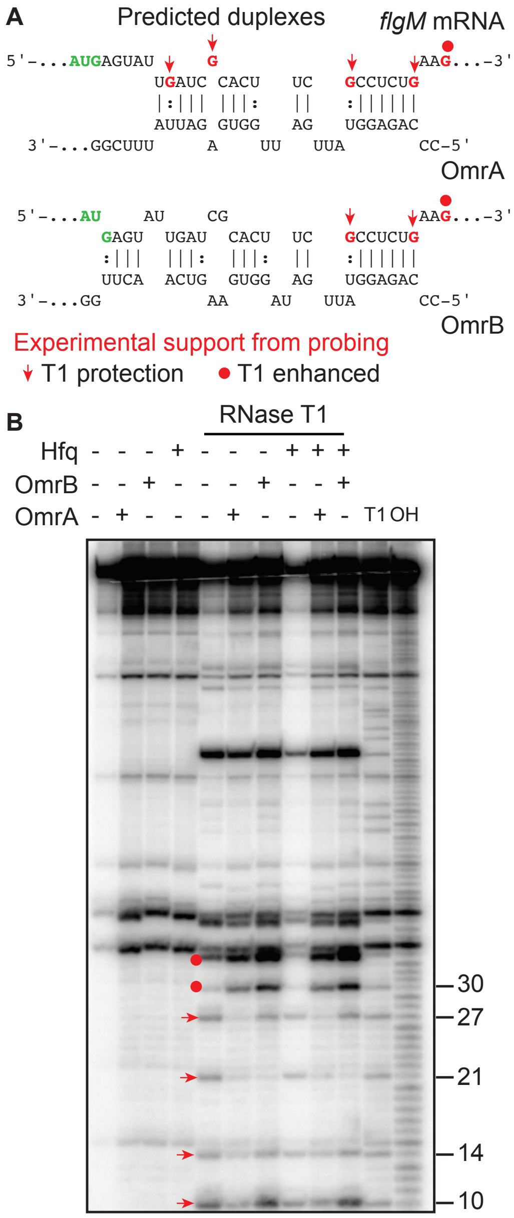

Figure S1. OmrA and B base-pair to *flgM* mRNA near the ribosome binding site.

A. Prediction of duplex formation between the *flgM* mRNA and OmrA or OmrB. The AUG start codon indicated is in green. G residues that show changes in cleavage intensity in the experiment displayed in B are highlighted in red; red dots - enhanced T1 cuts, red arrows - protection from T1 cleavage.

B. Footprinting experiment defining the OmrA/OmrB binding site on labeled *flgM* mRNA, in the absence or presence of Hfq. Protection against RNase T1 cleavage: red arrows; enhanced T1 cuts: red circles. Nucleotide positions, relative to the AUG start codon, are indicated next to the autoradiogram and are based on the T1 ladder (T1) and hydroxyl radical ladder (OH).

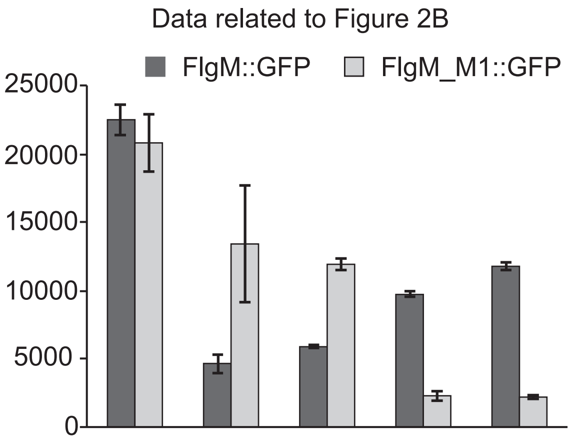
Data related to Figure 2B
FlgM::GFP
FlgM_M1::GFP
25000
20000
15000
10000
5000
0

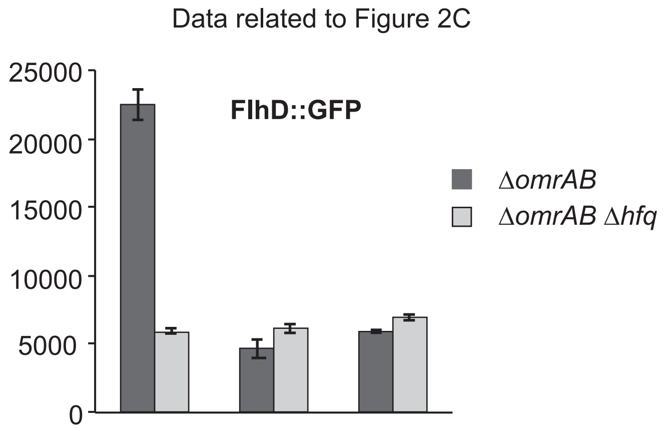
Data related to Figure 2C
FlhD::GFP
ΔomrAB
ΔomrAB Δhfq
25000
20000
15000
10000
5000
0

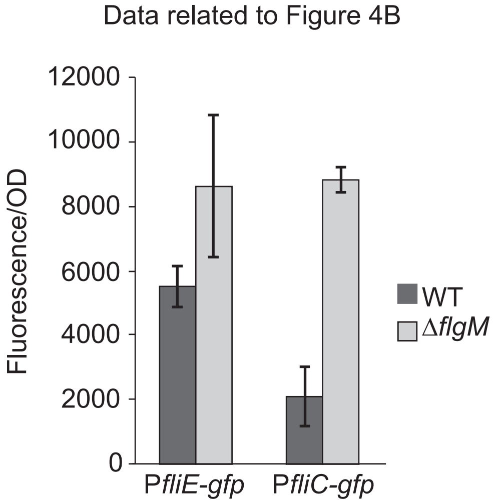
Data related to Figure 4B
Fluorescence/OD
12000
10000
8000
6000
4000
2000
0
WT
ΔflgM
PfliE-gfp
PfliC-gfp

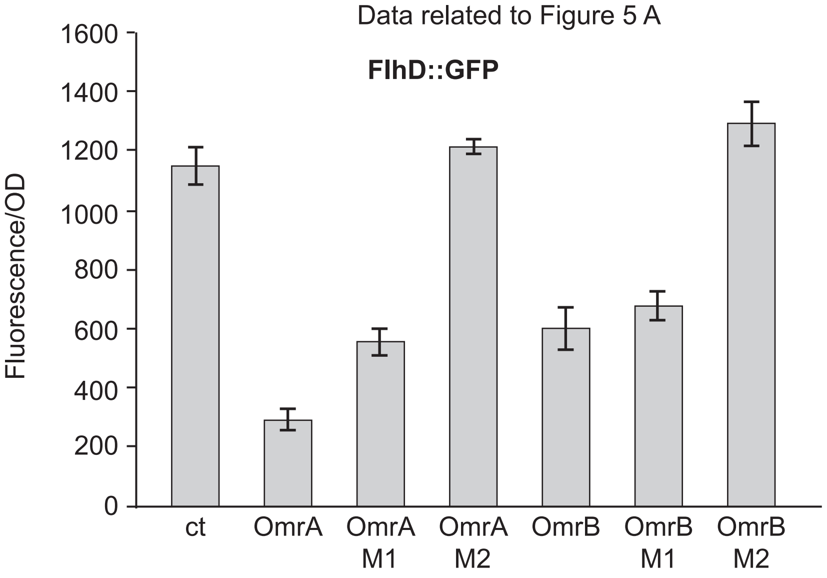
Data related to Figure 5 A
FlhD::GFP
Fluorescence/OD
1600
1400
1200
1000
800
600
400
200
0
ct
OmrA
OmrA M1
OmrA M2
OmrB
OmrB M1
OmrB M2

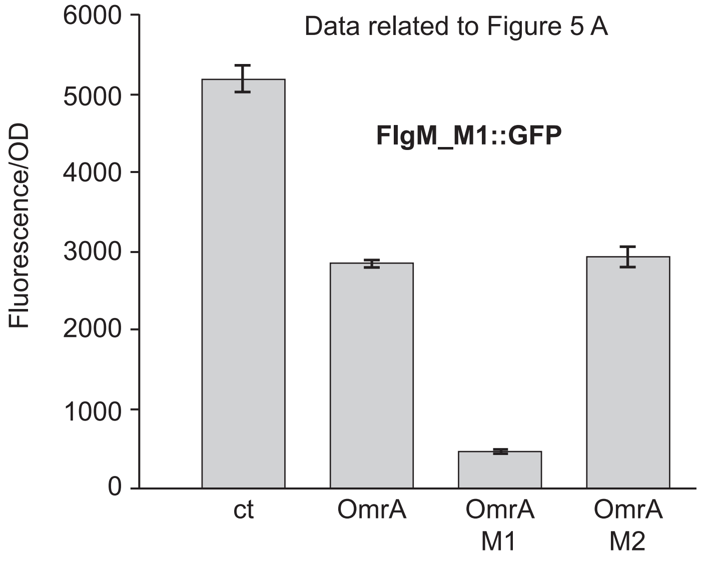
Data related to Figure 5 A
FlgM_M1::GFP
Fluorescence/OD
6000
5000
4000
3000
2000
1000
0
ct
OmrA
OmrA M1
OmrA M2

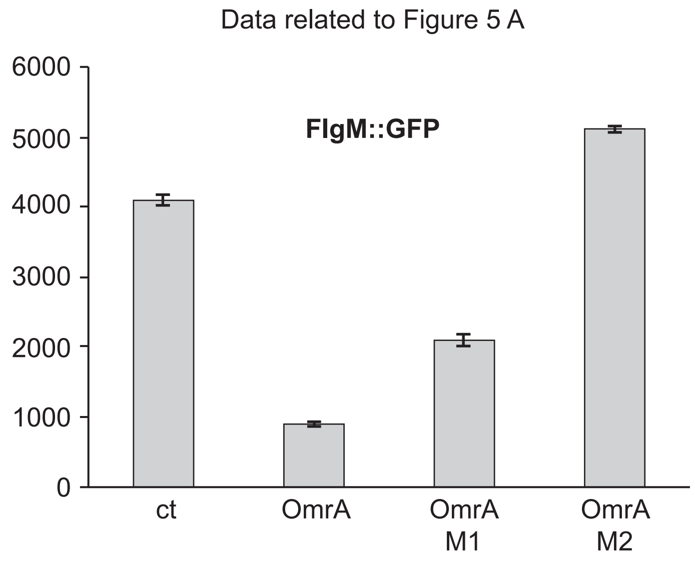
Data related to Figure 5 A
FlgM::GFP
6000
5000
4000
3000
2000
1000
0
ct
OmrA
OmrA M1
OmrA M2

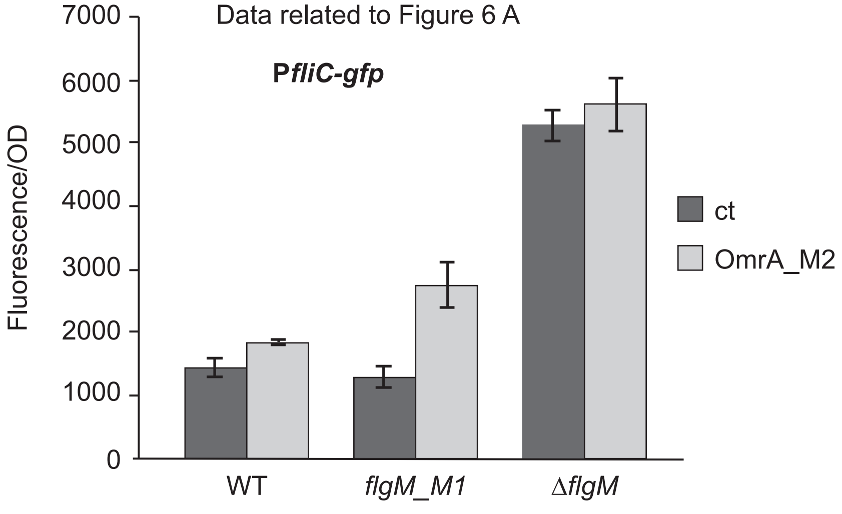
Data related to Figure 6 A
PfliC-gfp
Fluorescence/OD
7000
6000
5000
4000
3000
2000
1000
0
ct
OmrA_M2
WT
flgM_M1
ΔflgM

flhD mRNA

```
   5'-...ACAUC           U  A       C    AAAUAA        UA       UGGGA...-3'
              ACGGGGUGCGG GA    ACCG AUAA       AGUUGGU  UUCUGGG
              ||:||||:||: ||    |||| ||||       |:|:::|  :||||||
              UGUCCCAUGCU CU    UGGC UAUU       UUAGUUA  GAGACCC
   3'-...UUCUC           U  CGCA    U    AGAGUGG       UG       -5'
```

OmrA

flhD mRNA

```
   5'-...ACAUC           U  A       C    AAAUAA        UA       UGGGA...-3'
              ACGGGGUGCGG GA    ACCG AUAA       AGUUGGU  UUCUGGG
              ||:||||:||: ||    |||| ||||       |:|:::|  :||| ||
              UGUCCCAUGCU CU    UGGC UAUU       UUAGUUA  GAGAGCC
   3'-...UUCUC           U  CGCA    U    AGAGUGG       UG       -5'
```

OmrA_M1

flhD mRNA

```
   5'-...ACAUC           U  A       C    AAAUAA        UA       UGGGA...-3'
              ACGGGGUGCGG GA    ACCG AUAA       AGUUGGU  UUCUGGG
              ||:||||:||: ||    |||| ||||       |:|:::|  :|||  |
              UGUCCCAUGCU CU    UGGC UAUU       UUAGUUA  GAGAGGC
   3'-...UUCUC           U  CGCA    U    AGAGUGG       UG       -5'
```

OmrA_M2

flgM mRNA

```
5'-...AUGAGUAU     G                   AAG...-3'
              UGAUC CACU   UC   GCCUCUG
              |:||| |||:   ||   :||||||
              AUUAG GUGG   AG   UGGAGAC
  3'-...GGCUUU     A    UU   UUA       CC-5'
```

OmrA

flgM_M1 mRNA

```
5'-...AUGAGUAU     G                   AAG...-3'
              UGAUC CACU   UC   GCCUCUC
              |:||| |||:   ||   :||||||
              AUUAG GUGG   AG   UGGAGAG
  3'-...GGCUUU     A    UU   UUA       CC-5'
```

OmrA_M1

flgM_M1 mRNA

```
5'-...AUGAGUAU     G                   AAG...-3'
              UGAUC CACU   UC   GCCUCUC
              |:||| |||:   ||   :||||||
              AUUAG GUGG   AG   UGGAGAG
  3'-...GGCUUU     A    UU   UUA       GC-5'
```

OmrA_M2

Figure S3. Duplex prediction between the *flhD, flgM* or *flgM_M1* mRNAs with OmrA, OmrA_M1 or OmrA_M2

Table S1. Primers used in this study

Primer name	Sequence (5’ to 3’)[a,b]	Template	Remarks
ced287	GGAATAAACCATGAGTATTGATCGCACTTCGCCTCTGAAGTGTAGGCTGGAGCTGCTTC	ced58	PCR product for *kan-sacB* cassette insertion into the chromosome in the *flgM gene*
ced288	GCGCGTCAGTGGTTTCGCGCGGTTGAACGGTGCTTACAGGACATATGAATATCCTCCTTAGTTCC	ced58	PCR product for *kan-sacB* cassette insertion into the chromosome in the *flgM gene*
ced289	CCCTCAATGAGGAATAAACCATGAGTATTGATCGCACTTCGCAAGACTTGCAGAGTAACTGATCGTATGACACGTCTTGCAG	ced78	PCR product for in frame deletion of *flgM*
ced290	CTGCAAGACGTGTCATACGATCAGTTACTCTGCAAGTCTTGCGAAGTGCGATCAATACTCATGGTTTATTCCTCATTGAGGG	ced78	PCR product for in frame deletion of *flgM*
ced373	AACACATGATAAAAGCGCCCTCAATGAG	pflgM_M1::gfp	PCR product for point mutation in *flgM*
ced374	GCGTCAGTGGTTTCGCGCGG	pflgM_M1::gfp	PCR product for point mutation in *flgM*
ced291	AACCTATCCAGTTAACCCA	BW25113 / MC4100	Amplification of the *flgM* gene
ced292	AACTGTTCGAGGTAATCCA	BW25113 / MC4100	Amplification of the *flgM* gene
EHO-671	GTTTTT*ATGCAT*AACACATGATAAAAGCGCCCTCAATGAG	BW25113 / MC4100	PCR for flgM::GFP plasmid creation
EHO-433	GTTTTT*GCTAGC*GTCAGTGGTTTCGCGCGG	BW25113 / MC4100	PCR for flgM::GFP plasmid creation
EHO-696	CGCACTTCGCCTCTCAAGCCTGTAAGCAC	pflgM::gfp	PCR for flgM_M1::GFP plasmid creation
EHO-697	GTGCTTACAGGCTTGAGAGGCGAAGTGCG	pflgM::gfp	PCR for flgM_M1::GFP plasmid creation
EHO-698	CAAGATACTGAGCACCCGAGAGGTATTGATTGGTG	pOmrA	PCR for pOmrA_M1 plasmid creation
EHO-699	CACCAATCAATACCTCTCGGGTGCTCAGTATCTTG	pOmrA	PCR for pOmrA_M1 plasmid creation
EHO-700	CAAGATACTGAGCACCCGAGAGGTATTGATAGGTG	pOmrB	PCR for pOmrB_M1 plasmid creation
EHO-701	CACCTATCAATACCTCTCGGGTGCTCAGTATCTTG	pOmrB	PCR for pOmrB_M1 plasmid creation
EHO-363	CTGAGCACCGGAGAGGTATTGATTGGTGAGATTATT	pOmrA	PCR for pOmrA_M2 plasmid creation
EHO-364	AATAATCTCACCAATCAATACCTCTCCGGTGCTCAG	pOmrA	PCR for pOmrA_M2 plasmid creation
EHO-367	CTGAGCACCGGAGAGGTATTGATAGGTGAAGTCAAC	pOmrB	PCR for pOmrB_M2 plasmid creation
EHO-368	GTTGACTTCACCTATCAATACCTCTCCGGTGCTCAG	pOmrB	PCR for pOmrB_M2 plasmid creation
EHO-348	GAAAT**TAATACGACTCACTATAG**GGAGATGGGCTGATGAGTCCGTGAGGACGAAACGGTACCCGGTACCGTCCCCAGAGGTATTGATTGG	EHO-349	For T7-Hammerhead-OmrA, anneal to EHO-349, fill-in
EHO-349	AAAAAAAACCTGCGCATCCGCGCAGGTTGGTGCAAGAGACAGGGTACGAAGAGCGTACCGAATAATCTCACCAATCAATACCTCTGGGGA	EHO-348	For T7-Hammerhead-OmrA, anneal to EHO-348, fill-in
EHO-350	GAAAT**TAATACGACTCACTATAG**GGAGATGGGCTGATGAGTCCGTGAGGACGAAACGGTACCCGGTACCGTCCCCAGAGGTATTGATAG	EHO-351	For T7-Hammerhead-OmrB, anneal to EHO-351, fill-in
EHO351	AAAAAAAACCTGCGCATCTGCGCAGGCTGGTGTAATTCATGTGCTCAACCCGAAGTTGACTTCACCTATCAATACCTCTGGGGACGG	EHO-350	For T7-Hammerhead-OmrB, anneal to EHO-350, fill-in
MHO-200	GCCGGGTTGCATCAGTTTTG	*flgM* mRNA / MC4100	Toeprint oligo and *flgM* mRNA transcription
EHO-714	GAAATT**AATACGACTCACTATAG**GGAACACATGATAA AAGCGCCCTCAA	MC4100	With MHO 200, *flgM* mRNA transcription
MHO-207	GG**TAATACGACTCACTATAG**GCCAGGGGTGCTCGGCATAA	E397	*ompA 3xflag* mRNA transcription
MHO-230	ACGAAAGTCAGTTCAATTTACTAAAGGC	E397	*ompA 3xflag* mRNA transcription
MHO-238	GTTTTT*CTCGAG*CCCTATAGTGAGTCGTATTAATTTCG	pET52b (Novagen)	Linearization of pET52b, removal His tag, addition of 3xFLAG and XhoI/AatII sites
MHO-239	GTTTTT*GACGTC*GACTACAAAGACCATGACGGTGATTATAAAGATCATGATATCGACTACAAAGATGACGACGATAAATAGTAAATTAACCTAGGCTGCTGCCA	pET52b (Novagen)	Linearization of pET52b, removal His tag, addition of 3xFLAG and XhoI/AatII sites
MHO-240	GTTTTTCTCGAGAACACATGATAAAAGCGCCCTCAA	MC4100	PCR of 5’UTR + flgM CDS minus stop codon, and addition of XhoI/AatII sites
MHO-241	GTTTTTGACGTCGTTACTCTGCAAGTCTTGCTGCG	MC4100	PCR of 5’UTR + flgM CDS minus stop codon, and addition of XhoI/AatII sites
MHO-244	TACCCACGCCGAAACAAG	pET52b – T7 –flgM-3xflag	*flgM 3xflag* mRNA transcription
MHO-245	AGCAAAAAACCCCTCAAGAC	pET52b – T7 –flgM-3xflag	*flgM 3xflag* mRNA transcription

(a) **TAATACGACTCACTATAG**: T7 promoter sequence (b) *NNNNNN* Restriction site

Table S2. Strains used in this study

Strain name	Relevant genotype	Plasmid 1	Plasmid 2	Reference	Remarks
	MC4100 *relA+ ΔomrAB*	-	-	(Holmqvist et al., 2010)	
	BW25113	-	-	*E. coli* stock collection	
ced58	MG1655 *rpsA::kan-SacB*	-	-	(Romilly et al., 2019)	Template to obtain the kan-sacB cassette
MH268	BW25113 ΔomrAB::FRT-tet-FRT	-	-	This study	
ced74	MC4100 *relA+ ΔomrAB*	pSim5	-	This study	Scarless mutagenesis
ced78	MC4100 relA+ *ΔomrAB flgM::kan-sacB*	pSim5	-	This study	Scarless mutagenesis, kan-sacB cassette insertion, with PCR product obtained with ced287/ced288 primers
ced75	BW25113	pSim5	-	This study	Scarless mutagenesis
ced79	BW25113 *flgM::kan-sacB*	pSim5	-	This study	Scarless mutagenesis, kan-sacB cassette insertion, with PCR product obtained with ced287/ced288 primers
ced81	MC4100 relA+ Δ*flgM* Δ*omrAB*	-	-	This study	In-frame deletion, scarless mutagenesis from ced78, with PCR product obtained with ced289/ced290 primers
ced84	BW25113 Δ*flgM*	-	-	This study	In-frame deletion, scarless mutagenesis from ced79, with PCR product obtained with ced289/ced290 primers
ced118	BW25113 Δ*flgM* Δ*omrAB::FRT-tet-FRT*	-	-	This study	P1 transduction, MH268 donor, ced84 recipient
ced158	BW25113 *flgM_M1*	-	-	This study	Point mutation, scarless mutagenesis from ced79, with PCR product obtained with ced373/ced374 primers
ced159	BW25113 *flgM_M1* Δ*omrAB::FRT-tet-FRT*	-	-	This study	P1 transduction, MH268 donor, ced158 recipient
E922	MC4100 *relA+ ΔomrAB*	pflgM::GFP	pControl	This study	Used for data in Fig.2, flgM translational fusion
E923	MC4100 *relA+ ΔomrAB*	pflgM::GFP	pOmrA	This study	Used for data in Fig.2, flgM translational fusion
E924	MC4100 *relA+ ΔomrAB*	pflgM::GFP	pOmrB	This study	Used for data in Fig.2, flgM translational fusion
E968	MC4100 *relA+ ΔomrAB*	pflgM::GFP	pOmrA_M1	This study	Used for data in Fig.2, flgM translational fusion
E969	MC4100 *relA+ ΔomrAB*	pflgM::GFP	pOmrB_M1	This study	Used for data in Fig.2, flgM translational fusion
E965	MC4100 *relA+ ΔomrAB*	pflgM_M1::GFP	pControl	This study	Used for data in Fig.2, flgM_M1 translational fusion
E966	MC4100 *relA+ ΔomrAB*	pflgM_M1::GFP	pOmrA	This study	Used for data in Fig.2, flgM_M1 translational fusion
E967	MC4100 *relA+ ΔomrAB*	pflgM_M1::GFP	pOmrB	This study	Used for data in Fig.2, flgM_M1 translational fusion
E970	MC4100 *relA+ ΔomrAB*	pflgM_M1::GFP	pOmrA_M1	This study	Used for data in Fig.2, flgM_M1 translational fusion
E971	MC4100 *relA+ ΔomrAB*	pflgM_M1::GFP	pOmrB_M1	This study	Used for data in Fig.2, flgM_M1 translational fusion
E1009	MC4100 *relA+ ΔomrAB Δhfq (FRT-tet-FRT)*	pflgM::GFP	pControl	This study	Used for data in Fig.2, flgM translational fusion
E1010	MC4100 *relA+ ΔomrAB Δhfq (FRT-tet-FRT)*	pflgM::GFP	pOmrA	This study	Used for data in Fig.2, flgM translational fusion
E1011	MC4100 *relA+ ΔomrAB Δhfq (FRT-tet-FRT)*	pflgM::GFP	pOmrB	This study	Used for data in Fig.2, flgM translational fusion
ced129	BW25113 Δ*omrAB::FRT-tet-*FRT	pfliC	-	This study	Used for data in Fig.4, *fliC* transcriptional fusion
ced130	BW25113 Δ*omrAB::FRT-tet-*FRT	pfliE	-	This study	Used for data in Fig.4, *fliE* transcriptional fusion
ced131	BW25113 Δ*flgM* Δ*omrAB::FRT-tet-*FRT	pfliC	-	This study	Used for data in Fig.4, *fliC* transcriptional fusion
ced132	BW25113 Δ*flgM* Δ*omrAB::FRT-tet-FRT*	pfliE	-	This study	Used for data in Fig.4, *fliE* transcriptional fusion
ced120	MC4100 *relA+* Δ*flgM* Δ*omrAB*	pflhD::GFP	pControl	This study	Used for data in Fig.5, flhD::GFP translational fusion
ced121	MC4100 *relA+* Δ*flgM* Δ*omrAB*	pflhD::GFP	pOmrA	This study	Used for data in Fig.5, flhD::GFP translational fusion
ced122	MC4100 *relA+* Δ*flgM* Δ*omrAB*	pflhD::GFP	pOmrB	This study	Used for data in Fig.5, flhD::GFP translational fusion
ced123	MC4100 *relA+* Δ*flgM* Δ*omrAB*	pflhD::GFP	pOmrA_M1	This study	Used for data in Fig.5, flhD::GFP translational fusion
ced124	MC4100 *relA+* Δ*flgM* Δ*omrAB*	pflhD::GFP	pOmrB_M1	This study	Used for data in Fig.5, flhD::GFP translational fusion
ced125	MC4100 *relA+* Δ*flgM* Δ*omrAB*	pflhD::GFP	pOmrB_M2	This study	Used for data in Fig.5, flhD::GFP translational fusion
ced126	MC4100 *relA+* Δ*flgM* Δ*omrAB*	pflhD::GFP	pOmrA_M2	This study	Used for data in Fig.5, flhD::GFP translational fusion

ced175	MC4100 *relA*+ Δ*omrAB*	pflgM_M1::GFP	pControl	This study	Used for data in Fig.5, flgM_M1::GFP translational fusion
ced176	MC4100 *relA*+ Δ*omrAB*	pflgM_M1::GFP	pOmrA	This study	Used for data in Fig.5, flgM_M1::GFP translational fusion
ced177	MC4100 *relA*+ Δ*omrAB*	pflgM_M1::GFP	pOmrA_M1	This study	Used for data in Fig.5, flgM_M1::GFP translational fusion
ced178	MC4100 *relA*+ Δ*omrAB*	pflgM_M1::GFP	pOmrA_M2	This study	Used for data in Fig.5, flgM_M1::GFP translational fusion
ced169	BW25113 Δ*omrAB::FRT-tet*-FRT	pfliC	pControl	This study	Used for data in Fig. 6, *fliC* transcriptional fusion
ced170	BW25113 Δ*omrAB::FRT-tet*-FRT	pfliC	pOmrA_M2	This study	Used for data in Fig. 6, *fliC* transcriptional fusion
ced171	BW25113 *flgM_M1* Δ*omrAB::FRT-tet-FRT*	pfliC	pControl	This study	Used for data in Fig. 6, *fliC* transcriptional fusion
ced172	BW25113 *flgM_M1* Δ*omrAB::FRT-tet-FRT*	pfliC	pOmrA_M2	This study	Used for data in Fig. 6, *fliC* transcriptional fusion
ced173	BW25113 Δ*flgM* Δ*omrAB::FRT-tet-FRT*	pfliC	pControl	This study	Used for data in Fig. 6, *fliC* transcriptional fusion
ced174	BW25113 Δ*flgM* Δ*omrAB::FRT-tet-FRT*	pfliC	pOmrA_M2	This study	Used for data in Fig. 6, *fliC* transcriptional fusion
E397	MC4100 relA+ ompA 3xFLAG	-	-	(Holmqvist et al., 2010)	Used for ompA mRNA T7 transcription
ced189	MC4100 *relA*+ Δ*omrAB*	pflgM_M1::GFP	pControl	This study	Used for data in Fig.5, flgM:GFP translational fusion
ced190	MC4100 *relA*+ Δ*omrAB*	pflgM_M1::GFP	pOmrA	This study	Used for data in Fig.5, flgM::GFP translational fusion
ced191	MC4100 *relA*+ Δ*OMRab*	pflgM_M1::GFP	pOmrA_M1	This study	Used for data in Fig.5, flgM::GFP translational fusion
ced192	MC4100 *relA*+ Δ*omrAB*	pflgM_M1::GFP	pOmrA_M2	This study	Used for data in Fig.5, flgM::GFP translational fusion

Holmqvist, E., Reimegård, J., Sterk, M., Grantcharova, N., Römling, U., and Wagner, E.G.H. (2010). Two antisense RNAs target the transcriptional regulator CsgD to inhibit curli synthesis. EMBO J. *29*, 1840–1850.

Romilly, C., Deindl, S., and Wagner, E.G.H. (2019). The ribosomal protein S1-dependent standby site in tisB mRNA consists of a single-stranded region and a 5′ structure element. Proc. Natl. Acad. Sci. USA, 116, 15901-15906.

Table S3. Plasmids used in this study

Plasmid name	Stock name	Reference	Remarks
pflgM::GFP	pEH159	This study	Translational fusion
pflgM_M1::GFP	pEH173	This study	Translational fusion
pControl	pJV300	(Sittka et al., 2007)	Empty control plasmid
pOmrA	pEH67	(Holmqvist et al., 2010)	Constitutive expression of OmrA
pOmrB	pEH68	(Holmqvist et al., 2010)	Constitutive expression of OmrB
pOmrA_M1	pEH171	This study	Constitutive expression of OmrA_M!
pOmrB_M1	pEH172	This study	Constitutive expression of OmrB_M1
pOmrA_M2	pEH80	This study	Constitutive expression of OmrA_M2
pOmrB_M2	pEH83	This study	Constitutive expression of OmrB_M2
pflhD::GFP	pEH260	This study	Translational fusion
pfliC	pSC21-fliC	(Kalir et al., 2001)	Transcriptional fusion
pFliE	pSC21-fliE	(Kalir et al., 2001)	Transcriptional fusion
pflgM 3xFLAG	pMH26	This study	Template for PCR

Holmqvist, E., Reimegård, J., Sterk, M., Grantcharova, N., Römling, U., and Wagner, E.G.H. (2010). Two antisense RNAs target the transcriptional regulator CsgD to inhibit curli synthesis. EMBO J. *29*, 1840–1850.

Kalir, S., McClure, J., Pabbaraju, K., Southward, C., Ronen, M., Leibler, S., Surette, M.G., and Alon, U. (2001). Ordering Genes in a Flagella Pathway by Analysis of Expression Kinetics from Living Bacteria. Science *292*, 2080–2083.

Sittka, A., Pfeiffer, V., Tedin, K., and Vogel, J. (2007). The RNA chaperone Hfq is essential for the virulence of Salmonella typhimurium. Mol. Microbiol. *63*, 193–217.
